# PFKFB3-mediated glycometabolism reprogramming modulates endothelial differentiation and angiogenic capacity of placenta-derived mesenchymal stem cells

**DOI:** 10.1186/s13287-022-03089-3

**Published:** 2022-08-02

**Authors:** Yang Zhang, Yanqi Zhong, Weifang Liu, Fanghui Zheng, Yin Zhao, Li Zou, Xiaoxia Liu

**Affiliations:** Department of Obstetrics and Gynecology, Union Hospital, Tongji Medical College, Huazhong University of Science and Technology, No. 1277 Jiefang Avenue, Wuhan, 430022 China

**Keywords:** Mesenchymal stem cells, Placenta, Endothelial differentiation, Glycometabolism reprogramming, Glycolysis, PFKFB3, Angiogenesis

## Abstract

**Background:**

Mesenchymal stem cells (MSCs) have a great potential ability for endothelial differentiation, contributing to an effective means of therapeutic angiogenesis. Placenta-derived mesenchymal stem cells (PMSCs) have gradually attracted attention, while the endothelial differentiation has not been fully evaluated in PMSCs. Metabolism homeostasis plays an important role in stem cell differentiation, but less is known about the glycometabolic reprogramming during the PMSCs endothelial differentiation. Hence, it is critical to investigate the potential role of glycometabolism reprogramming in mediating PMSCs endothelial differentiation.

**Methods:**

Dil-Ac-LDL uptake assay, flow cytometry, and immunofluorescence were all to verify the endothelial differentiation in PMSCs. Seahorse XF Extracellular Flux Analyzers, Mito-tracker red staining, Mitochondrial membrane potential (MMP), lactate secretion assay, and transcriptome approach were to assess the variation of mitochondrial respiration and glycolysis during the PMSCs endothelial differentiation. Glycolysis enzyme 6-phosphofructo-2-kinase/fructose-2,6-bisphosphatase 3 (PFKFB3) was considered a potential modulator for endothelial differentiation in PMSCs by small interfering RNA. Furthermore, transwell, *in vitro* Matrigel tube formation, and *in vivo* Matrigel plug assays were performed to evaluate the effect of PFKFB3-induced glycolysis on angiogenic capacities in this process.

**Results:**

PMSCs possessed the superior potential of endothelial differentiation, in which the glycometabolic preference for glycolysis was confirmed. Moreover, PFKFB3-induced glycometabolism reprogramming could modulate the endothelial differentiation and angiogenic abilities of PMSCs.

**Conclusions:**

Our results revealed that PFKFB3-mediated glycolysis is important for endothelial differentiation and angiogenesis in PMSCs. Our understanding of cellular glycometabolism and its regulatory effects on endothelial differentiation may propose and improve PMSCs as a putative strategy for clinical therapeutic angiogenesis.

**Supplementary Information:**

The online version contains supplementary material available at 10.1186/s13287-022-03089-3.

## Introduction

Angiogenesis, the formation of new blood vessels from preexisting vasculature, is closely involved in tissue and organ growth, development, and regeneration [1]. Angiogenesis dysfunction has been widely observed in multiple ischemic diseases, including but not limited to myocardial infarction, diabetic wounds, and preeclampsia [2]. Previously, angiogenic factors, oxygen regulation, pharmaceutical agents, and other therapeutic angiogenesis methods were clinically performed to enhance the angiogenic potential at the sites of injury [3]. Currently, researchers are gradually turning toward cell-based therapies [4]. Endothelial cells (ECs) are one of the direct components of the vessels; however, the obstacle to the therapeutic application of autologous ECs implantation is the difficulty in obtaining adequate ECs as well as the formation of a complex functional neovasculature [5]. Mesenchymal stem cells (MSCs), a group of multipotent, self-renewing, and undifferentiated cells, can differentiate into a variety of cell lineages such as adipocytes, osteoblasts, chondrocytes, and even endothelial cells (ECs) under appropriate conditions [6]. Thereinto, bone marrow-derived MSCs (BMMSCs) have long been considered the gold standard. BMMSCs can show vigorous induced endothelial differentiation both in vitro and in vivo while obtaining bone marrow has been a challenge [7, 8]. Therefore, hunting for new and versatile alternatives is necessary. Placenta, a transient fetomaternal organ, is discarded after delivery without invasive procedures, making it easily more available and ethically more favorable with a rich supply of MSCs [9]. The placenta is a highly vascular organ, and human placenta-derived mesenchymal stem cells (PMSCs), residing in the placental vascular niche [10], are less prone to osteogenic, adipogenic, and myogenic differentiation than BMMSCs but reveal strong endothelial conversion properties [10–13]. PMSCs may differentiate into primitive ECs to initiate nascent vascularization during the early placenta development [14–17]. Meanwhile, PMSCs secrete a plethora of bioactive molecules to promote angiogenesis [18, 19]. Hence, PMSCs may have superior potencies of endothelial differentiation as well as angiogenesis. Nonetheless, compared to the BMMSCs counterparts, PMSCs are far less understood and explored.

As the major pathways providing metabolic precursors for biosynthesis and adenosine triphosphate (ATP) production, glycolysis and mitochondrial oxidative phosphorylation (OXPHOS) are delicately tuned to ensure optimal resource and energy distribution. The reprogramming between glycolysis and mitochondrial OXPHOS is essential for stem cell differentiation [20, 21]. Generally, stem cells mainly depend on glycolysis for energy metabolism [22], while MSCs differentiation is always accompanied by metabolism reprogramming, which in turn modulates the differentiation process [23]. Concretely, when MSCs commit to osteogenic and adipogenic differentiation, glycolytic metabolism tends to shift into the mitochondrial OXPHOS to meet increased cellular energy demand. However, mitochondrial dysfunction impairs the mesenchymal lineage differentiation of MSCs [24, 25]. Therefore, the metabolic conversion and MSCs differentiation may be inextricably linked and interacted. Nevertheless, it is still unclear concerning glycometabolism reprogramming during the PMSCs endothelial differentiation as well as the implication.

In this study, we firstly promoted the differentiation of PMSCs into ECs through the method as previously reported [7, 26]. Then, we thoroughly evaluated the glycometabolism shift during the PMSCs endothelial differentiation. Lastly, ribonucleic acid sequencing (RNA-seq) was performed to identify the potential molecular modulators of metabolic reprogramming and angiogenic capacities in the PMSCs endothelial differentiation. This study may provide new frameworks for considering the therapeutic angiogenesis of PMSCs.

## Methods and materials

### PMSCs isolation, culture, and identification

This study was approved by the Ethics Institutional Review Board of Union Hospital, Tongji Medical College, Huazhong University of Science and Technology (Ethic Code: S042 and S2487). Written informed consents were also obtained. Based on previously described methods [19, 27, 28], PMSCs were isolated from term placentas collected from healthy donors who had gone through elective cesarean sections due to the breech position or previous history of cesarean section. The placental tissue was taken from the center area around the umbilical cord attachment site and was gently washed with preheated phosphate-buffered saline (PBS, pH 7.2 ± 0.1, Genome Sciences), minced into 1mm^3^ pieces, and digested with gentle shaking in 0.1 mg/mL type I collagenase (Gibco) for 60 min at 37 °C. The digested tissue was filtered through a cell strainer (100 μm). Mononuclear cells were obtained by lymphocyte isolation (Ficoll, tbdscience) and washed twice with PBS. Then, the sediment was resuspended in DMEM/F12 (Gibco) medium supplemented with penicillin, streptomycin, and 10% fetal bovine serum (FBS, Gibco). Eventually, the cells were plated in each T25 flask (Corning) at 1 × 10^6^ cells/mL and incubated at 37 °C with 5% CO_2_ for 72 h. Third-passage PMSCs were used for the subsequent experiments.

Surface phenotype analysis was evaluated by flow cytometry. Briefly, PMSCs were digested with 0.25% trypsin–EDTA (Gibco) and washed with PBS. Cell suspensions were incubated with PBS or fluorescein-labeled antibodies for 30 min. The tested antibodies included CD73, CD90, CD105, CD31, CD34, CD45, and HLA-DR (BD Biosciences, USA) as previously reported [19, 27, 28]. Isotype-identical antibodies were used as controls. The cells were acquired in a fluorescence activated-cell sorter (BD Biosciences, USA). Flow cytometric data were analyzed with Cell Quest-Pro software (BD Biosciences, USA).

Multilineage differentiation in PMSCs was also examined under adipogenic, chondrogenic, and osteogenic differentiation conditions [29]. PMSCs were seeded into six-well plates (Corning) at 4 × 10^5^ cells/well and cultured until they reached confluence. For adipogenic differentiation analysis, cells were incubated in the adipogenic differentiation medium (Cyagen Biosciences, USA) according to the manufacturer's instructions. The medium was changed every 3 days. After 21 days of induction, the cells were fixed in PBS containing 10% formaldehyde solution, stained with oil red O (Sigma-Aldrich, St. Louis, Mo), and imaged with an optical microscope (Olympus, JAPAN). For chondrogenic differentiation analysis, cells were treated with the chondrogenic differentiation medium (Cyagen Biosciences, USA) according to the manufacturer's instructions. The medium was changed every 3 days. After 21 days of induction, the cells were then stained with alcian blue (Sigma-Aldrich) and imaged with an optical microscope. For osteogenic differentiation analysis, cells were incubated in the osteogenic differentiation medium (Cyagen Biosciences, USA) according to the manufacturer's instructions. The medium was changed every 3 days. After 21 days of induction, the mineralized osteocytes were visualized with alizarin red (Sigma-Aldrich) staining and imaged with an optical microscope.

### Isolation and culture of human umbilical vein endothelial cells (HUVECs)

HUVECs were separated and identified following our previous report [30]. Briefly, umbilical cords got from term pregnancies without complications were purged with cold PBS. Then, 0.05% type I collagenase was injected into the umbilical veins. Incubated at 37 °C for 30 min, umbilical veins were washed with 20 mL PBS to collect HUVECs and then centrifugation at 288 × g for 5 min. The supernatant was discarded and cells were resuspended in ECM (ScienCell, USA). The cell suspension was then inoculated into T25 flasks and incubated at 37 °C with 5% CO_2_. After 3 days, the primary cells reached 80–90% confluence and then they were subcultured. HUVECs of passages 2–6 were used in our study.

### Endothelial differentiation of PMSCs and BMMSCs

The BMMSCs in this study were a kind gift from Prof. Cao Yang (Department of Orthopaedics, Union Hospital, Tongji Medical College, Huazhong University of Science and Technology, Wuhan, China). Approved by the Ethics Committee of Tongji Medical College, Huazhong University of Science and Technology (no. S347), they have harvested human bone marrow specimens from the ilia of healthy volunteer donors. Isolation and identification of human BMMSCs had been conducted as they had previously reported [31, 32]. BMMSCs were cultured in DMEM containing 15% FBS and 1% penicillin–streptomycin. The cells from the second or third passage were used in our study. Currently, the endothelial differentiation of BMMSCs has been reported and verified by Wang et al. [7, 26]. According to the method Wang et al. reported, inducing medium containing 50 ng/mL (vascular endothelial growth factor (VEGF, Lonza, Basel, Switzerland), 10 ng/mL basic fibroblast growth factor (bFGF, Lonza), 20 ng/mL epidermal growth factor (EGF, Lonza), 5 ng/mL insulin-like growth factor (IGF, Lonza), ascorbic acid, heparin, and 2% FBS was applied to PMSCs and BMMSCs and changed subsequently every day [7, 26].

To confirm the endothelial phenotype, the induced cells were harvested and analyzed for EC-specific markers (CD31 and CD34) by flow cytometry after 3, 7, 10, and 14 days to evaluate the presence of differentiated ECs [33]. Additionally, through immunofluorescence and flow cytometry, the uptake of DiI-Ac-LDL (Solarbio) was to verify the PMSCs differentiation toward endothelial phenotype as well [34].

Immunofluorescence staining was all done for von Willebrand factor (vWF) and vascular endothelial growth factor receptor-2 (VEGFR-2) to ensure the endothelial differentiation of PMSCs.

### Immunofluorescence staining

Immunofluorescence staining was performed on PMSCs in different groups. The cells were grown on poly-l-lysine-coated coverslips in 24-well culture plates. At 80% confluency, cells were fixed in 4% paraformaldehyde for 30 min, rinsed with PBS twice, blocked with 1% bovine serum albumin (BSA) for 30 min, and incubated with rabbit vWF antibody (11,778–1-AP, Proteintech), VEGFR-2 antibody (26,415–1-AP, Proteintech) in a humidified box at 4 °C overnight, followed by washing in PBS three times. FITC-conjugated goat anti-rabbit secondary antibody (Proteintech) or CY3-conjugated goat anti-rabbit secondary antibody (Proteintech) was then added and incubated for a further 30 min. Finally, samples were counterstained with DAPI (Beyotime) in antifade reagent and observed under confocal microscopy (Nikon AIR SI Confocal; Nikon). The control samples consisted of cells without primary antibodies and were used to assess the background fluorescence.

### Oxygen consumption rate (OCR) and extracellular acidification rate (ECAR)

For OCR and ECAR measurement, cells were seeded into Seahorse 24-well plates and were then treated according to the manufacturer’s protocol after the indicated treatment. Seahorse Xfe24 analyzer (Seahorse Bioscience, Boston, MA, USA) was used for evaluation. All results were normalized to the cell number. For OCR measurement, oligomycin, carbonylcyanide-p-trifluoromethoxyphenylhydrazone (FCCP), and rotenone were added. Basal respiration = (last rate measurement before oligomycin injection)—(minimum rate measurement after rotenone injection). Maximal respiration = (maximum rate measurement after FCCP injection)—(minimum rate measurement after rotenone injection). For ECAR evaluation, glucose, oligomycin, and 2-deoxyglucose (2-DG) were added according to the manufacturer’s instructions. Glycolysis = (last rate measurement before oligomycin injection)—(minimum rate measurement before glucose injection). Glycolytic capacity = (maximum rate measurement after oligomycin injection)—(minimum rate measurement after 2-DG injection).

### Mito‑tracker staining

Mitochondrial morphology under the confocal microscope was observed by Mito-tracker red staining (C1035, Beyotime) using a confocal microscope according to the previous research [35]. The length of mitochondria was determined by ImageJ software. At least 6 images per condition were analyzed.

### Mitochondrial membrane potential (MMP)

MMP was evaluated via fluorescent probe JC-1 (C2006, Beyotime) using a confocal microscope. Normal mitochondria were presented as red, while mitochondria with decreased MMP were presented as green. The ratio of green/red fluorescence intensity was calculated by ImageJ software to assess the MMP changes. At least 6 images per condition were analyzed. MMP was also evaluated by a flow cytometer (BD Biosciences Pharmingen). The cells with healthy mitochondria were distributed in Q2, while cells with MMP decline were distributed in Q3.

### Measurements of the lactate concentration

Extracellular lactate levels were measured using the lactate assay kit (A019-2–1, Nanjing Jiancheng), according to the manufacturer’s instructions.

### Total RNA extraction

Total RNA was extracted from the tissues using TRIzol (Invitrogen, Carlsbad, CA, USA) according to manual instruction. About 60 mg of tissues was ground into powder by liquid nitrogen in a 2-ml tube, followed by being homogenized for 2 min and rested horizontally for 5 min. The mix was centrifuged for 5 min at 12,000 × g at 4 °C, and then the supernatant was transferred into a new EP tube with 0.3 mL chloroform/isoamyl alcohol (24:1). The mix was shacked vigorously for 15 s and then centrifuged at 12,000 × g for 10 min at 4 °C. After centrifugation, the upper aqueous phase where RNA remained was transferred into a new tube with an equal volume of supernatant of isopropyl alcohol, then centrifuged at 13,600 rpm for 20 min at 4 °C. After deserting the supernatant, the RNA pellet was washed twice with 1 mL 75% ethanol, and then the mix was centrifuged at 13,600 rpm for 3 min at 4 °C to collect residual ethanol, followed by the pellet air-dry for 5–10 min in the biosafety cabinet. Finally, 25–100 µL of DEPC-treated water was added to dissolve the RNA. Subsequently, total RNA was qualified and quantified using a NanoDrop and Agilent 2100 Bioanalyzer (Thermo Fisher Scientific, MA, USA).

### mRNA library construction

Oligo(dT)-attached magnetic beads were used to purify mRNA. Purified mRNA was fragmented into small pieces with fragment buffer at an appropriate temperature. Then First-strand cDNA was generated using random hexamer-primed reverse transcription, followed by second-strand cDNA synthesis. Afterward, A-Tailing Mix and RNA Index Adapters were added by incubating to end repair. The cDNA fragments obtained from the previous step were amplified by PCR, and products were purified by AMPure XP Beads and then dissolved in EB solution. The product was validated on the Agilent Technologies 2100 Bioanalyzer for quality control. The double-stranded PCR products from the previous step were heated denatured and circularized by the splint oligo sequence to get the final library. The single-strand circle DNA (ssCir DNA) was formatted as the final library. The final library was amplified with phi29 to make DNA nanoball (DNB) which had more than 300 copies of one molecular, DNBs were loaded into the patterned nanoarray and single end 50 bases reads were generated on the BGIseq500 platform (BGI-Shenzhen, China).

### Ribonucleic acid sequencing data processing and identification of differentially expressed genes

cDNA libraries from MSCs and induced ECs were sequenced according to the protocols for RNA-Seq. Raw reads were preprocessed using FastQC software. PCR duplicates, reads that only contain adapter, ploy-N, and reads with low quality (score ≤ 5) were removed. Clean reads were then used for subsequent analyses. Transcript expression levels were estimated using fragments per kilobase per million reads (FPKM) values and quantified by RSEM software. EdgeR software was used to identify differentially expressed genes. The package employed robust statistical models even for small numbers of replicates.

### Gene Ontology and Kyoto Encyclopedia of Genes and genomes enrichment analysis

For functional enrichment analysis, all differentially expressed genes (DEGs) were mapped to terms in the Gene Ontology (GO) databases, and then significantly enriched GO terms were searched for among the DEGs using *P* < 0.05 as the threshold. GO term analysis was classified into three subgroups, namely biological process (BP), cellular component (CC), and molecular function (MF). All DEGs were mapped to the KEGG database and searched for significantly enriched KEGG pathways at *P* < 0.05 level.

### Quantitative real-time quantitative polymerase chain reaction (qRT-PCR)

The primer sequences were as follows:

- β-actin (internal group) forward primer: 5′-CACGATGGAGGGGCCGGACTCATC-3′; reverse primer: 5′-TAAAGACCTCTATGCCAACACAGT-3′.
- LDHD forward primer: 5′-CGCACAGAGGAGATAGTCCA-3′; reverse primer: 5′-CAGATCCTCCTTGGTCTGCAC -3′.
- PFKFB2 forward primer: 5′-AGTCCTACGACTTCTTTCGGC-3′; reverse primer: 5′-TCTCCTCAGTGAGATACGCCT-3′.
- PFKFB3 forward primer: 5′-ATTGCGGTTTTCGATGCCAC-3′; reverse primer: 5′- GCCACAACTGTAGGGTCGT-3′.
- PFKFB4 forward primer: 5′-ACAGTGATGAGGCTACGG-3′; reverse primer: 5′- ATGCGGCTCTGGATGTG-3′.
- HK2 forward primer: 5′-GAGTTTGACCTGGATGTGGTTGC -3′; reverse primer: 5′- CCTCCATGTAGCAGGCATTGCT -3′.

qRT-PCR was performed on StepOne Real-Time PCR System with SYBR Premix Ex Taq (Vazyme) as described in the manufacturer’s instructions. The qRT-PCR reaction consisted of a 95 °C denaturation step for 30 s, 40 cycles (95 °C for 5 s, 65 °C for 30 s, and 60 °C for 45 s), and an extension at 72 °C for 60 s. The expression of target genes was established using the comparative cycle threshold (Ct; 2 ^− ΔΔCT^) method.

### RNA interference

PFKFB3 siRNA and scrambled siRNA were synthesized by GenePharma Co., Ltd. (GenePharma, Shanghai, China). The previously validated siRNA sequence targeting PFKFB3 was shown below: 5′-AGUUGUAGGAGCUGUACUG-3′. siRNAs (100 nM) were introduced into cultured cells with HiPerfect transfection reagent (Qiagen) after reaching 70% confluence [36]. The transfected PMSCs were used for endothelial differentiation after 48 h. The silencing effects of siRNAs were confirmed by RT-PCR analysis after 48 h transfection.

### Conditioned medium preparation

After PMSCs were grown in complete medium until 80% confluent, the culture medium of PMSCs in different groups was removed. Cell layers were washed with PBS twice and subsequently incubated with serum-free DMEM/F12 for 24 h at 37℃, 5% CO2. Then, the conditioned medium was collected, centrifuged at 2000 rpm for 10 min, filtered through a 0.22-μm filter, and stored at − 80 °C for the following experiments.

### Cell migration assay

Transwell units (24-well plates, 8-μm pores; Corning Costar, NY, USA) were investigated for the cell migration capacities. Different groups of PMSCs were resuspended in 200 μL of serum-free medium and placed in the upper chamber, and 500 μL complete medium was added to the lower chamber. The transwell units were incubated at 37℃, 5% CO2 for 24 h. The units were stained with crystal violet for 30 min; then, the cells were removed from the upper-membrane surface. Six fields were chosen randomly and the number of the underside of the membrane was counted under a microscope. Collected conditioned medium of different groups of PMSCs was added with 10% FBS additionally and then was used to pretreat HUVECs for 24 h. The following migration steps were consistent with those mentioned above.

### In vitro tube formation assay

Aliquots of 50 μl Matrigel (Corning) were plated into each well of 96-well plates (Corning) and polymerized for 30 min at 37 °C. The PMSCs labeled with CMFDA (cell tracker green Molecular Probes), EC-differentiated PMSCs labeled with CMFDA, and HUVECs labeled with CMTPX (cell tracker red, Molecular Probes) were resuspended at 10^6^ cells/ml. The cell suspension (100 μl) was added, respectively, to each well and incubated at 37 °C for 4 h.

For tube formation assay in the HUVECs and PMSCs coculture system, 50 μl CMFDA-labeled PMSCs or CMFDA-labeled EC-differentiated PMSCs suspension, together with 50 μl CMTPX-labeled HUVECs suspension, was added to each well and incubated at 37 °C for 4 h. The tube formation was visualized under phase-contrast microscopy (Olympus, Tokyo, Japan) and the ratio of green/red fluorescence intensity was calculated by ImageJ software.

### In vivo Matrigel plug assays

All animal studies were approved by the Ethic Institutional Review Board of Union Hospital, Tongji Medical College, Huazhong University of Science and Technology (Ethic Code: S2487). Matrigel (500 µL) containing PBS, PMSCs, Endothelium-differentiated PMSCs, siPFKFB3-Endothelium-differentiated PMSCs, HUVECs, PMSCs + HUVECs, endothelium-differentiated PMSCs + HUVECs, or siPFKFB3-Endothelium-differentiated PMSCs + HUVECs was injected subcutaneously into the abdomen of nude mice (n = 3). The total cell number was 5 × 10^6^ cells/gel, and the ratio of each kind of PMSCs to HUVECs was 3:2. The Matrigel was removed 12 days after transplantation and prepared for paraffin sections. Post-preparation, sections were stained with hematoxylin and eosin (H&E) for morphometric analysis. The blood vessels were stained with human-specific CD31 antibody (11,265–1-AP, Proteintech) and examined by microscope (Olympus, Lake Success, NY, USA) [37].

### Immunohistochemistry

The procedures for immunohistochemical detection were as follows: First, for antigen retrieval, rehydrated paraffin sections were heated in a citrate buffer (pH, 6.0) for 15 min. Then, the endogenous peroxidase activity was quenched by incubating the sections in 3% H_2_O_2_ for 20 min and blocking the sections with 10% normal goat serum for 1 h. Next, the sections were incubated with primary antibody (CD31, 1:200, 11,265–1-AP, Proteintech) at room temperature for 1 h. Lastly, the slides were incubated with horseradish peroxidase‐conjugated goat anti-rabbit IgG (1:1,000; Santa Cruz, Dallas, TX) for 30 min and peroxidase reactivity was detected using a diaminobenzidine substrate kit.

### Statistical analyses

For all analyses, data are shown as the mean ± standard deviation. The Student’s t test was used for comparing two samples, and multi-group comparison was carried out using one-way ANOVA followed by Tukey’s post hoc test. Statistical analysis was performed using GraphPad Prism8.0 (USA). The p value of less than 0.05 was considered statistically significant. All data were obtained from ≥ 3 independent experiments.

## Results

### Culture, identification, and endothelial differentiation of PMSCs

**Fig. 1:**
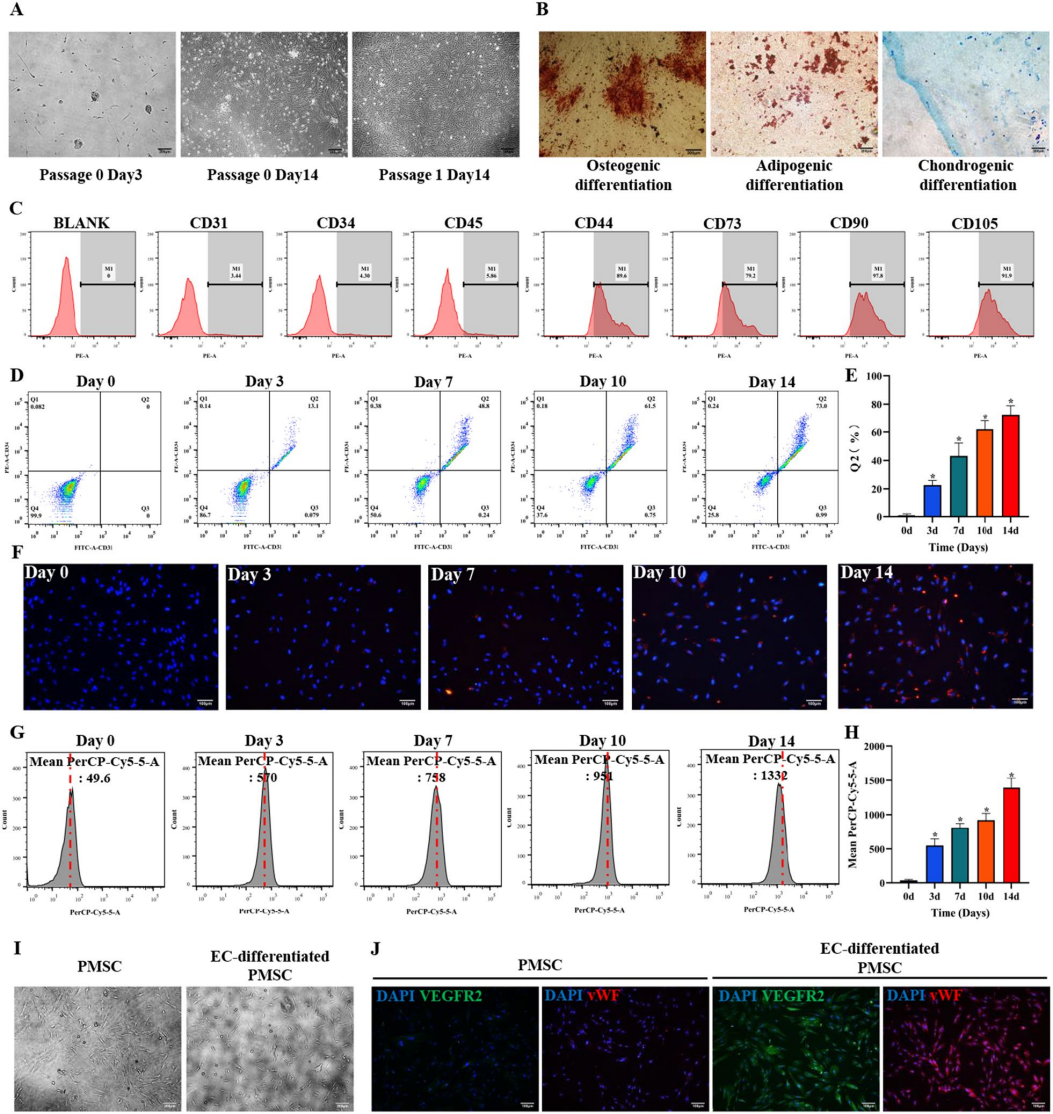
Culture, identification, and endothelial differentiation of PMSCs. **A** PMSCs morphology was detected by an inverted microscope. Three and seven days after isolation, small fibroblast-like MSC colonies were visible. Cell culture was enriched in a population of cells characterized by a fibroblast-like spike appearance. Scale bar: 200 μm. **B** Successfully differentiated PMSCs were stained with Alizarin Red for osteogenic differentiation, Oil Red O for adipocytes, and Alcian blue, in which red calcium nodules, orange lipid droplets, and blue cartilage could be observed. Scale bar: 200 μm. **C** PMSCs surface markers. Flow cytometry demonstrated that the cells expressed CD44, CD73, CD90, and CD105 but not CD31, CD34, and CD45. **D**, **E** Identification of induced PMSCs by flow cytometry and quantitative analyses were also performed. The fractions of CD31^+^ CD34^+^ double-positive cells were about 10%, 50%, 60%, and 70%, at 3, 7, 10, and 14 days, respectively. **F–H** Dil-Ac-LDL uptake assay was to identify the induced PMSCs through immunofluorescence and flow cytometry, and quantitative analyses were also performed. Scale bar: 100 μm. **I** Morphological characteristics of cells after cultivated in the inducing and non-inducing groups. The cells developed into short spindle shapes in the inducing group. The cells in the non-inducing medium retained the typical spindle-shaped morphology. Scale bar: 200 μm. **J** Immunofluorescence staining of vWF and VEGFR-2 was performed in different groups. Scale bar: 100 μm. Data are shown as the mean ± SD from three independent experiments and the representative result is shown. *P < 0.05, **P < 0.01, ***P < 0.001 by Student’s t test. SD: standard deviation. EC-differentiated PMSC: the induced PMSCs group for endothelial differentiation

Isolated PMSCs demonstrated a fibroblast-like, spindle-shaped morphology (Fig. 1A). The multipotency of PMSCs was detected via osteogenic, adipogenic, and chondrogenic differentiation (Fig. 1B). PMSCs expressed common MSCs markers (CD44, CD73, CD90, and CD105) but not CD31, CD34, CD45 (Fig. 1C) [38].

During the period of endothelial induction, the flow cytometry analysis monitored a gradual increase in endothelial markers (CD31 and CD34) expression on the 3rd, 7th, 10th, and 14th days (Fig. 1D, E). On the 3rd day of endothelial induction, about 10% of cells in Q2 quadrant were found CD31 and CD34 double-positive. The percentage of CD31^+^CD34^+^ cells increased to about 50% on the 7th day and about 60% in Q2 quadrant on the 10th day. The expression of CD31 and CD34 was strongly positive on the 14th day, with about 70% of cells observed in Q2 quadrant showing a positive expression (Fig. 1D, E). No expression of endothelial markers (CD31 and CD34) was observed at the whole cell culture stage in uninduced PMSCs. Also, the endothelial differentiation of PMSCs was confirmed by the Dil-Ac-LDL uptake assay, which was also increased in the whole endothelial induction (Fig. 1F, H). Ultimately, on the 14th day, PMSCs still retained the typical spindle-shaped morphology but developed into short spindle shapes during the endothelial differentiation (Fig. 1I). Immunofluorescence staining demonstrated that expression of vWF and VEGFR-2 was significantly higher in the induced PMSCs group compared with that in the uninduced PMSCs group (Fig. 1J). All these data above indicated the successful mesenchymal to endothelial differentiation in PMSCs.

### Glycometabolism reprogramming during the endothelial differentiation of PMSCs and BMMSCs

**Fig. 2:**
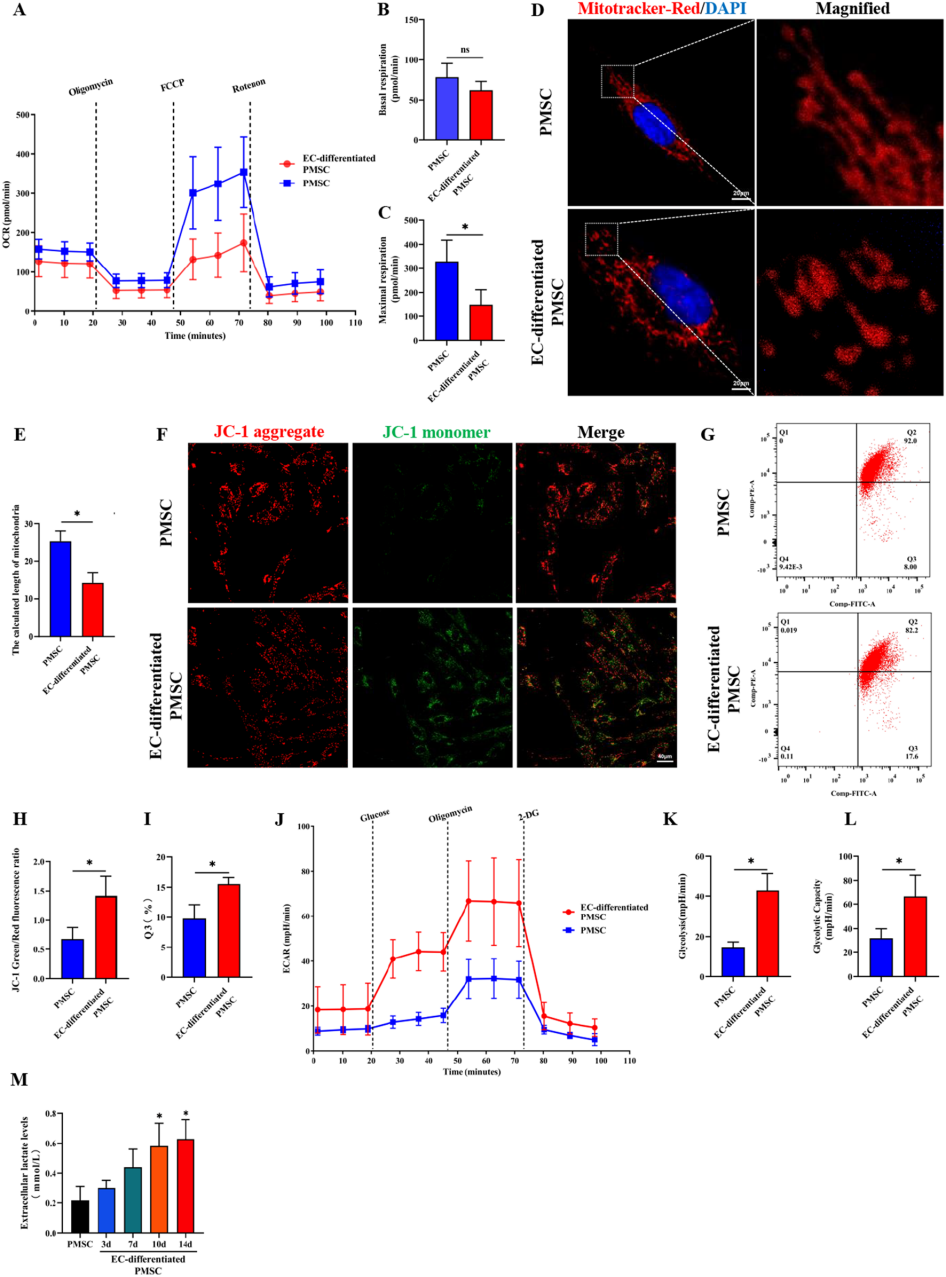
Glycometabolism reprogramming during the endothelial differentiation of PMSCs. **A–C** The OCR assay was used to observe the basal and maximal mitochondrial respiratory function. **D**, **E** Cells in different groups were stained with Mito-tracker red to evaluate the mitochondrial fragmentation, and the mitochondrial length was analyzed quantitatively. Scale bar: 20 μm. **E–I** MMP was evaluated by a confocal microscope and flow cytometry. Thereinto, the ratio of green/red puncta was calculated to assess the MMP changes, and quantitative analyses were also performed. Scale bar: 20 μm. **J–L** Glycolysis and glycolysis capacity was detected by ECAR assay. **M** Extracellular lactate release determination in PMSCs and endothelial differentiated PMSCs at Day 3, 7, 10, and 14. Data are shown as the mean ± SD from three independent experiments and the representative result is shown. *P < 0.05, **P < 0.01, ***P < 0.001 by Student’s t test. SD: standard deviation. EC-differentiated PMSC: the induced PMSCs group for endothelial differentiation

On the 14th day of endothelial induction, subsequent experiments were performed. OCR assays of OXPHOS were conducted, indicating that the OXPHOS was suppressed to some degree during the endothelial differentiation of PMSCs (Fig. 2A) and BMMSCs (Additional file 1: Fig. S1A). Compared to the undifferentiated state, the basal respiration rates of the endothelial differentiated PMSCs showed a reduced trend without significance (Fig. 2B), while the maximum respiration rates were significantly decreased (Fig. 2C). Similarly, the basal respiration rates and maximum respiration rates were both decreased in the induced BMMSCs (Additional file 1: Fig. S1B&C). Mitochondrial fission was quantified with the mitochondrial length [35], which was long, cable-like, thin strips in uninduced PMSCs. The mitochondria were divided into several short fragments during the endothelial differentiation of PMSCs (Fig. 2D, E). Mitochondrial membrane potential (MMP) assays via JC-1 fluorescence staining showed that the MMP significantly decreased during the endothelial differentiation of PMSCs (Fig. 2F, H). Consistent results were also obtained from the JC-1 flow cytometry analysis (Fig. 2G, I), suggesting the mitochondrial disturbance in this process. Measurement of ECAR revealed a rapid increase in glycolysis and glycolysis capacity in the differentiated group compared with the PMSCs (Fig. 2J–L), and the same results were found in the BMMSCs endothelial differentiation (Additional file 1: Fig. S1D&F). Besides, lactate secretion levels were significantly increased during the endothelial differentiation of PMSCs (Fig. 2M). All those above-mentioned data suggested the glycometabolic reprogramming to glycolysis in the PMSCs endothelial differentiation.

### Global identification of target genes modulating the glycometabolic conversion during the endothelial differentiation of PMSCs

**Fig. 3:**
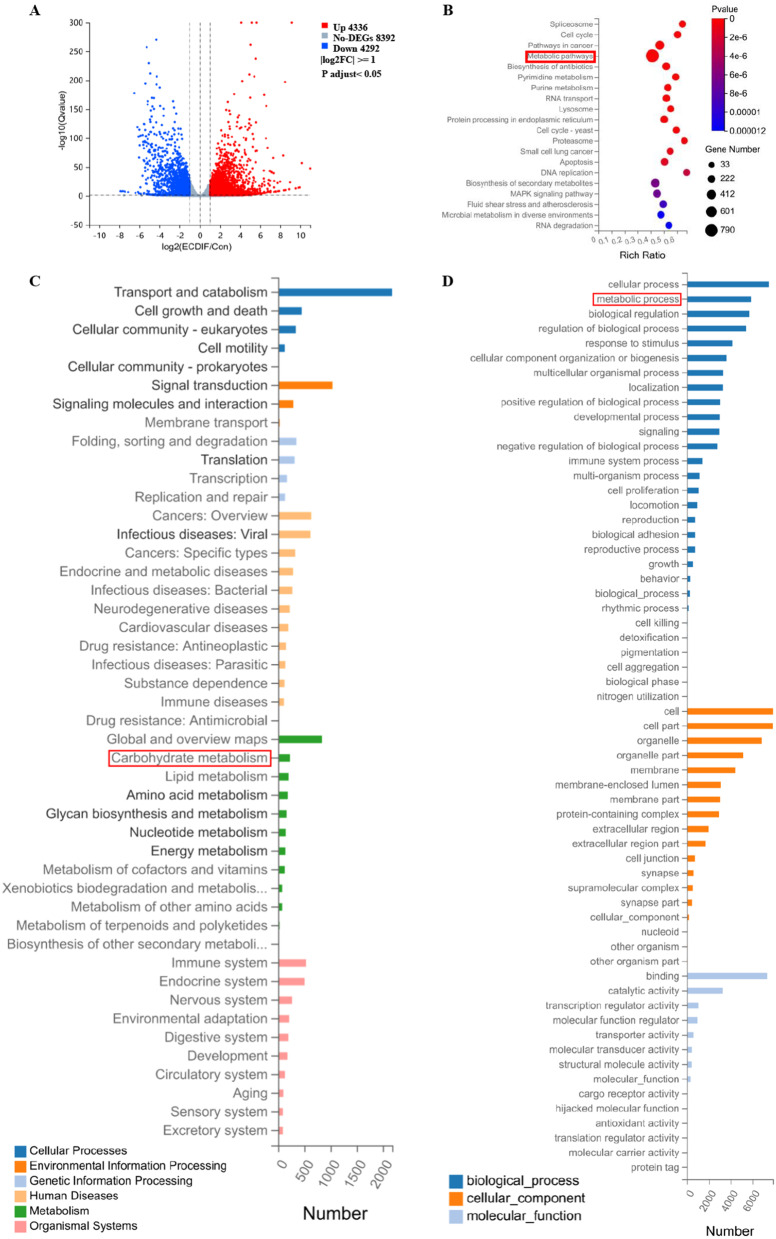
Bioinformatics analysis of whole transcriptome sequencing. **A** Volcano map of differentially expressed genes (DEGs) between PMSCs and induced PMSCs for endothelial differentiation. The x-axis is the log2 scale of the fold change of gene expression in MSCs and induced ECs (log2(fold change)). Negative values indicate downregulation; positive values indicate upregulation. The y-axis is the minus log10 scale of the adjusted *p* values (–log10 (p adj)), which indicates the significant level of expression difference. The red dots represent significantly upregulated genes, while the green dots represent significantly downregulated genes. **B** KEGG pathway enrichment analysis. **C** KEGG pathway classification analysis. **D** GO classification analysis. EC-differentiated PMSC: the induced PMSCs group for endothelial differentiation

On the 14th day of endothelial induction, subsequent experiments were performed. We performed RNA-seq transcriptomic analysis to identify the potential molecular mediators of metabolic reprogramming in the PMSCs endothelial differentiation. Specifically, endothelial differentiation induction significantly upregulated 4336 genes (log_2_(fold change) ≥ 1; adjusted *P* < 0.05) and suppressed 4292 genes (log_2_(fold change) ≤ -1; adjusted *P* < 0.05; Fig. 3A). Cell cycle and metabolism genes/pathways were highly enriched in the top 10 KEGG Pathway enrichment analysis (Fig. 3B). KEGG pathway classification analysis (Fig. 3C) and GO classification analysis (Fig. 3D) both indicated that most DEGs were annotated to the metabolism process such as the carbohydrate metabolism.

**Fig. 4:**
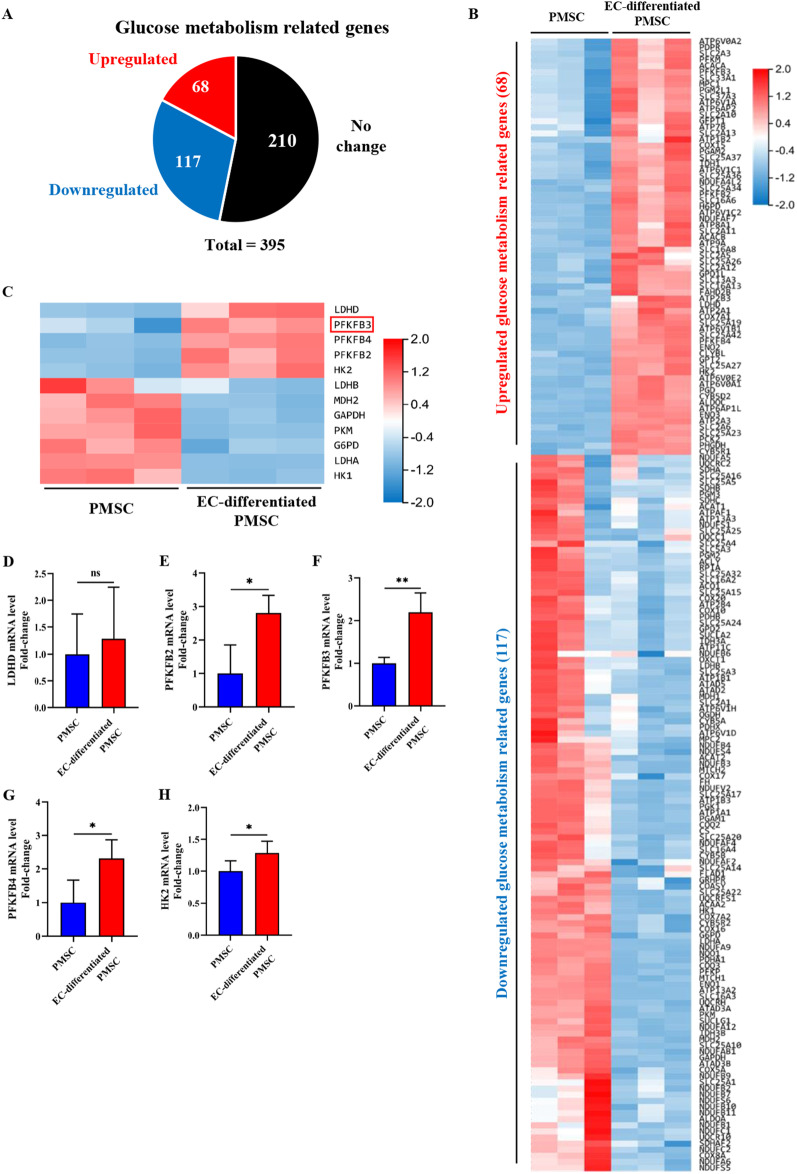
Global identification of target genes modulating the glycometabolic conversion during the endothelial differentiation of PMSCs. **A** Pie graph from RNA sequencing (RNA-seq) showing the expression profile of glucose metabolism-related genes in induced PMSCs for endothelial differentiation, with upregulated glucose metabolism-related genes and downregulated glucose metabolism-related genes. **B** The upregulated genes and downregulated genes from RNA-seq analysis. Color scales represent fold changes relative to control cells. **C** Heat maps of glycometabolic genes in induced PMSCs for endothelial differentiation. Color scales in heat maps represent fold changes relative to the uninduced PMSCs. **D–H** Quantitation from quantitative RT-PCR (real-time polymerase chain reaction) assays of the glycolysis genes LDHD, HK2, PFKFB2, PFKFB3, and PFKFB4. Data are shown as the mean ± SD from three independent experiments and the representative result is shown. *P < 0.05, **P < 0.01, ***P < 0.001 by Student’s t test. SD: standard deviation. EC-differentiated PMSC: the induced PMSCs group for endothelial differentiation

To understand the changes of glycometabolic genes in the endothelial differentiation of PMSCs, we next characterized a subset of 395 glucose metabolism-related genes based upon a published list of 1629 mammalian metabolic enzymes [39] and their protein functions using the UniProtKB database. Among this subset, 68 (log_2_(fold change) ≥ 1; adjusted *P* < 0.05) and 117 (log_2_(fold change) ≤ − 1; adjusted *P* < 0.05) genes were significantly stimulated or repressed (Fig. 4A, B), respectively, of which ATP2A3 (ATPase sarcoplasmic/endoplasmic reticulum Ca^2+^ transporting 3) was the most significantly upregulated (165-fold) and ATAD2 (ATPase family AAA domain-containing protein 2) was the most significantly downregulated (89.4%; Fig. 4B). Consistent with the enhanced glycolytic activity, the representative glycolytic genes HK2, PFKFB2, PFKFB3, PFKFB4, PGAM2, and PCK2 were significantly upregulated (Fig. 4B). Furthermore, we also observed significant reduction in mitochondrial respiratory complex genes such as NDUFA9, NDUFA12, COQ3, COQ2, COX8A (Fig. 4B), which was accompanied by marked increase in the transcripts encoding proteins with inhibitory effects on mitochondrial respiratory activity, such as SLC25A27 (Fig. 4B) [40]. These gene expression profiles are consistent with the suppressed activity of mitochondrial OXPHOS mentioned above. To validate these transcriptomic findings, we picked the mRNA expression of 12 key glucose metabolic genes (Fig. 4C). Consistent with RNA-seq findings, quantitation from quantitative RT-PCR (real-time polymerase chain reaction) assays also showed that the glycolysis genes LDHD HK2, PFKFB2, PFKFB3, and PFKFB4 were all upregulated (Fig. 4D–H). PFKFB3, also termed placental PFK-2, is well known for the highest kinase activity than bisphosphatase activity (700-fold), thus most favoring the catalytic activity to glycolysis [41, 42]. Besides, our research group has reported that PFKFB3 plays a vital role in some pathophysiological changes in the placentas [36, 43], including placental vasculature formation [44]. Given that the features and function of MSCs tend to be affected by the tissues from which MSCs are isolated [45], glycolytic enzyme PFKFB3 was chosen to be the putative modulator in the subsequent experiments.

### Glycolytic enzyme PFKFB3 regulated the endothelial differentiation of PMSCs

**Fig. 5:**
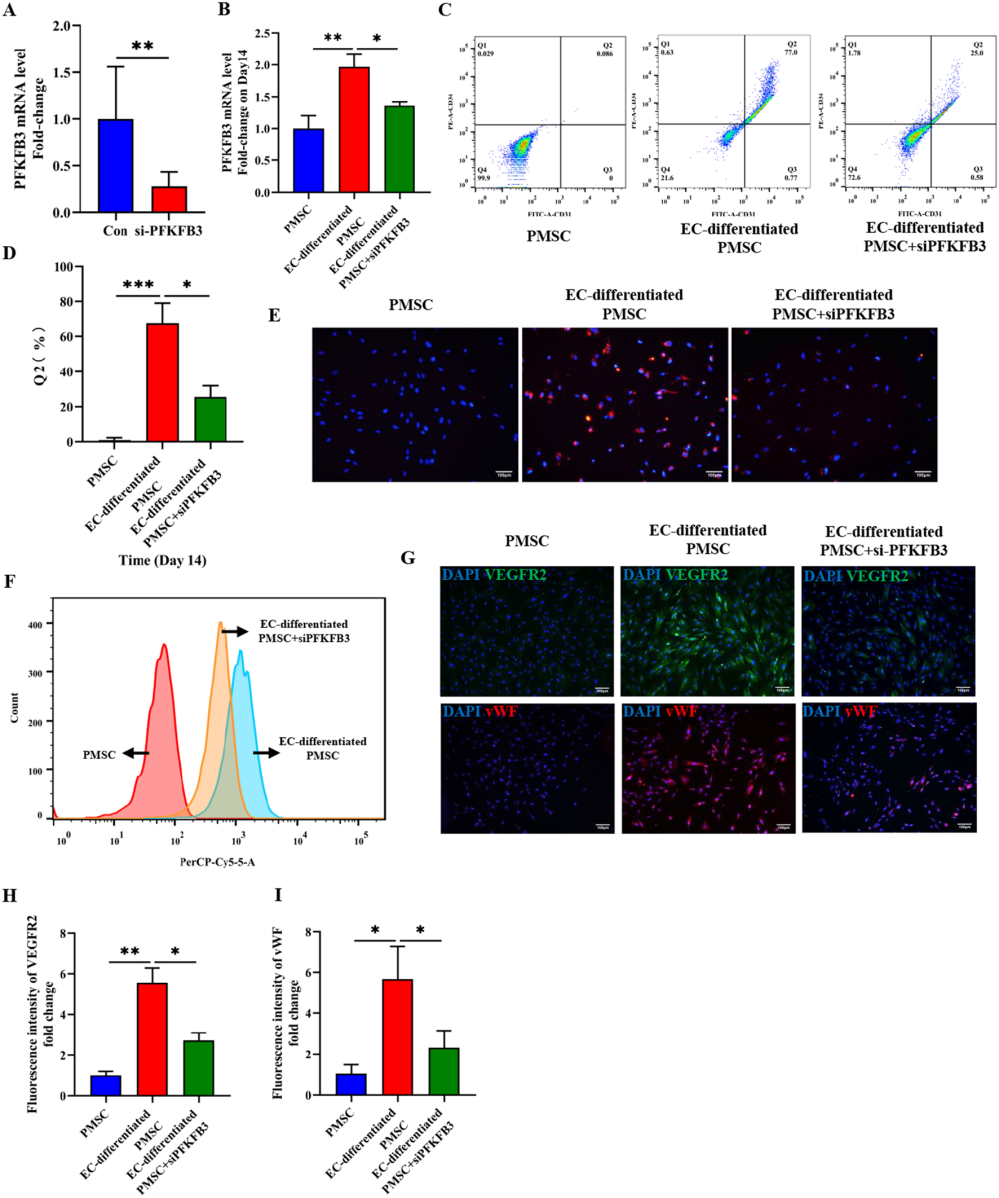
Glycolytic enzyme PFKFB3 regulated the endothelial differentiation of PMSCs. **A** Confirmation of PFKFB3 knockdown by qRT-PCR. (**B**) Detection of PFKFB3 mRNA level on the 14th day of endothelial induction. **C**, **D** flow cytometry and quantitative analyses of the fractions of CD31^+^CD34^+^ double-positive cells in different groups on Day 14. **E–F** Dil-Ac-LDL uptake assay through immunofluorescence and flow cytometry, and quantitative analyses in different groups on Day 14. Scale bar: 100 μm. **G** Immunofluorescence staining of vWF and VEGFR-2 in different groups. **H**, **I** Quantitative analysis of VEGFR-2 and vWF fluorescence intensity. Scale bar: 100 μm. Data are shown as the mean ± SD from three independent experiments and the representative result is shown. *P < 0.05, **P < 0.01, ***P < 0.001 by Student’s t test. SD: standard deviation. EC-differentiated PMSC: the induced PMSCs group for endothelial differentiation. si-PFKFB3: PFKFB3 siRNA

Figure 5A shows that PFKFB3 was successfully knocked down by the PFKFB3 siRNA, which is consistent with our previous report [36]. Figure 5B shows the detection of PFKFB3 mRNA level on the 14th day of endothelial induction in different groups, the result of which might ensure the stable inhibition of PFKFB3 along the endothelial differentiation process. The knockdown of PFKFB3 led to the downregulation of the endothelial biomarkers (CD31 and CD34) (Fig. 5C, D) and Dil-Ac-LDL uptake (Fig. 5E, F) on the 14^th^ day of endothelial induction. Also, immunofluorescence staining all showed that genetic inhibition of PFKFB3 significantly reduced the expression of vWF and VEGFR-2 on the 14th day of endothelial induction (Fig. 5G–I).

### PFKFB3 modulated the angiogenic and proangiogenic capacities of PMSCs during the endothelial differentiation in vitro and in vivo

**Fig. 6:**
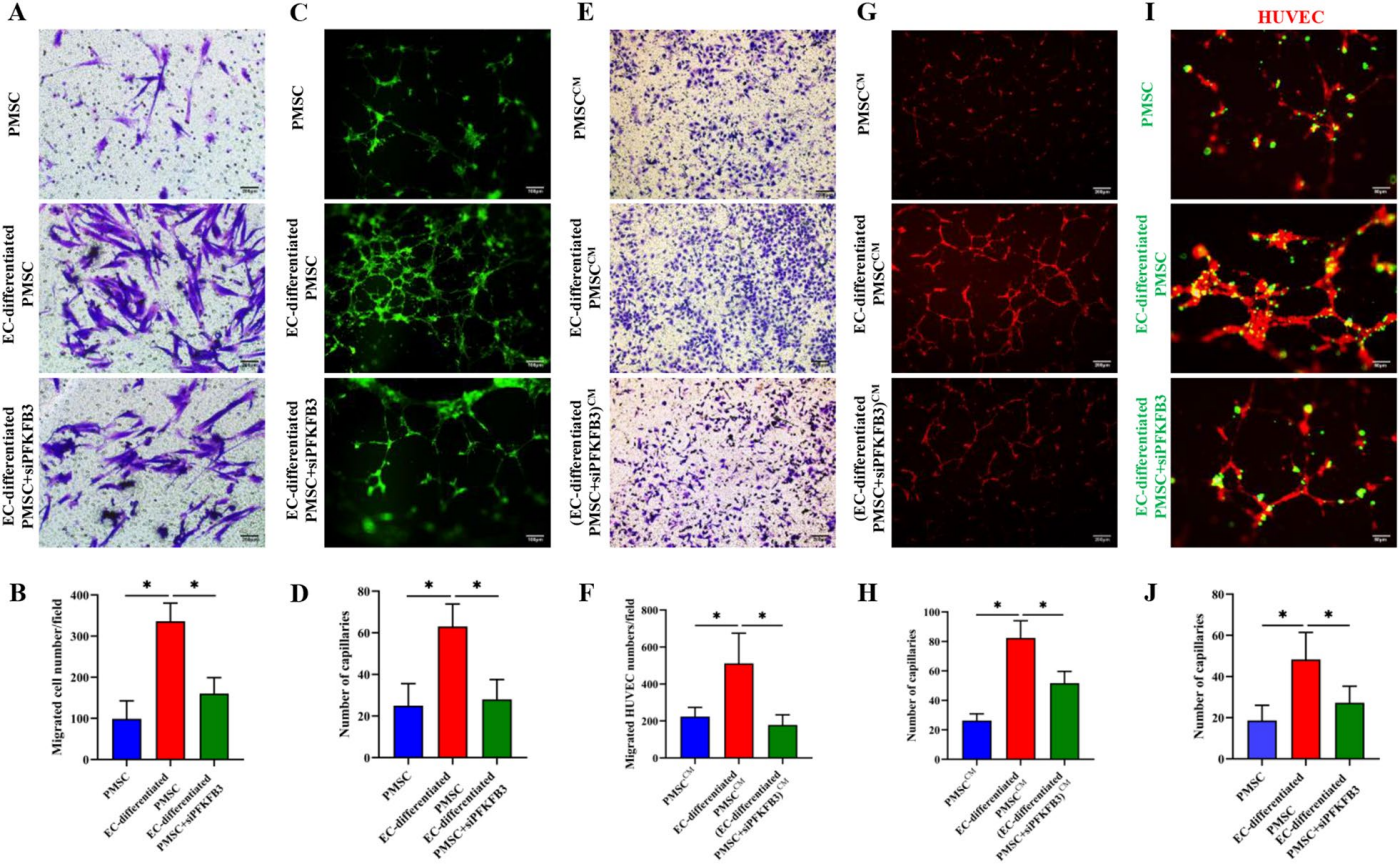
PFKFB3 modulated the angiogenic and proangiogenic capacities of PMSCs during the endothelial differentiation in vitro*.* **A**, **B** Representative transwell photographs of the PMSCs migration and the migrated PMSCs number quantified in different groups (Scale bar: 200 μm). **C**, **D** Representative photographs of the PMSCs tube formation and the number of formative capillaries quantified in different groups (Scale bar: 100 μm). **E**, **F** Representative transwell photographs of the HUVEC migration and the migrated HUVEC number quantified in different groups (Scale bar: 200 μm). **G**, **H** Representative photographs of the HUVEC tube formation and the number of formative capillaries quantified in different groups (Scale bar: 200 μm). **I**, **J** Representative photographs of the tube formation in the PMSC-HUVEC coculture system and the number of formative capillaries quantified in different groups (Scale bar: 50 μm). Data are shown as the mean ± SD from three independent experiments and the representative result is shown. *P < 0.05, **P < 0.01, ***P < 0.001 by Student’s t test. SD: standard deviation. EC-differentiated PMSC: the induced PMSCs group for endothelial differentiation. si-PFKFB3: PFKFB3 siRNA

**Fig. 7:**
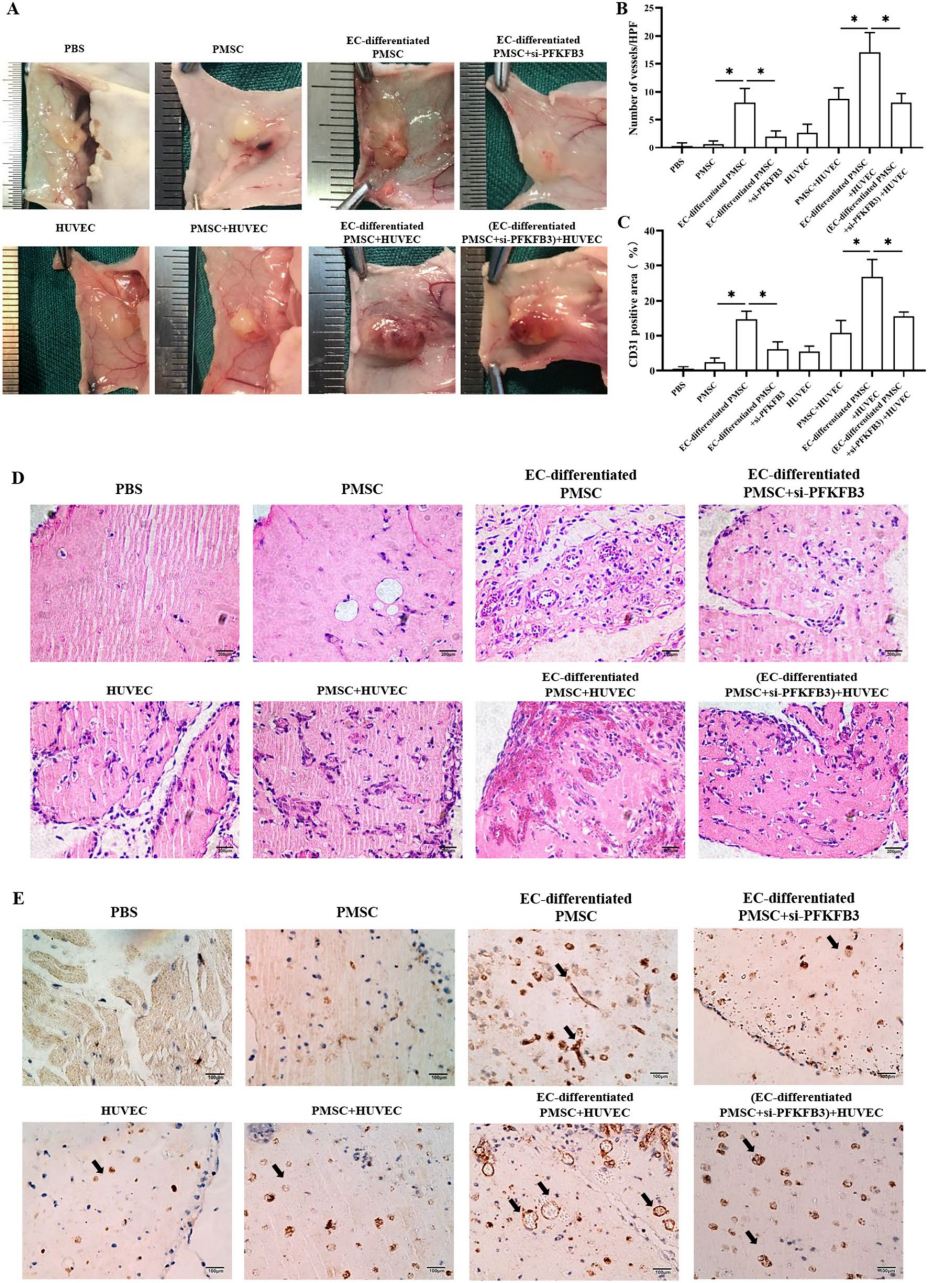
PFKFB3 modulated the angiogenic and proangiogenic capacities of PMSCs during the endothelial differentiation in Matrigel plugs in vivo. **A** Representative images of Matrigel plugs in different groups. **B–C** Quantitation of vessel number from the H&E-stained paraffin sections and quantitation of the human-specific CD31-positive percentage. **D**, **E** Representative images of macroscope view of explanted Matrigel plugs, H&E staining (Scale bar: 200 μm), and immunobiological human CD31 staining (Scale bar: 100 μm) of explanted Matrigel plugs. Data are shown as the mean ± SD from three independent experiments and the representative result is shown. *P < 0.05, **P < 0.01, ***P < 0.001 by Student’s t test. SD: standard deviation. EC-differentiated PMSC: the induced PMSCs group for endothelial differentiation. si-PFKFB3: PFKFB3 siRNA

On the 14th day of endothelial induction, subsequent experiments were performed. Compared to the undifferentiated PMSCs, the induced PMSCs showed better migratory ability (Fig. 6A, B) and tube formation capacity (Fig. 6C, D), while PFKFB3 siRNA suppressed those effects. After the endothelial induction of PMSCs, the cell-conditioned media significantly enhanced the migration and vessel formation of HUVECs, which was partly reversed via the genetic inhibition of PFKFB3 (Fig. 6E–H). Similarly, the *in vivo* Matrigel plug assays indicated the same results (Fig. 7A–E). In the HUVECs and PMSCs coculture system, *in vitro* tube formation assay on Matrigel (Fig. 6I, J) and *in vivo* Matrigel plug assays (Fig. 7A–E) both showed that the induced PMSCs more actively participated in the tube formation and increased vessel numbers, while the PFKFB3 siRNA inhibited those effects. The data above indicated the endothelial differentiation significantly augmented the angiogenic and proangiogenic potencies in PMSCs in vitro and in vivo, which was modulated via the glycometabolism reprogramming.

## Discussion

MSCs from multiple sources show the potential of endothelial differentiation in vivo and in vitro [46], among which BMMSCs are used to be the primary choice for therapeutic angiogenesis; however, it is difficult to acquire bone marrow, together with the inevitable ethical issue. PMSCs have now gained increasing attention for their abundance, ease of availability, low immunogenicity [47], effective immunomodulation [48], long-term growth [49], and potent expansion [50]. Despite the above-mentioned advantages, relatively little is understood about the endothelial differentiation in PMSCs. In this investigation, we promoted the endothelial differentiation in PMSCs and assessed the glycometabolic reprogramming in this differentiation process. We demonstrated that the glycometabolism conversion might interact with endothelial differentiation, in which glycolytic enzyme PFKFB3 might modulate the differentiation process and angiogenic capacities. Our understanding of cellular glycometabolism and its modulatory effects on endothelial differentiation may facilitate implementation in clinical therapeutic angiogenesis via improving the endothelial differentiation of PMSCs.

Oswald et al. firstly reported that VEGF induction (50 ng/mL, 1 week) induced endothelial differentiation of BMMSCs [51]; however, the only VEGF supplement or combination of VEGF and bFGF failed to induce endothelial differentiation in most cases, implying that VEGF and bFGF treatment may not be an effective and stable induction [7]. EGF contributes to vessel formation [52] and IGF increases the number of newly formed microvessels [53]. Wang et al. have introduced that a cocktail of cytokines (VEGF, bFGF, EGF, and IGF) can significantly raise the ratio of differentiated ECs from BMMSCs up to 60% after 2 weeks. Thus, the differentiation system supplemented with multiple cytokines might be more efficient than the single or two proangiogenic factors [7]. Lu et al. have conducted endothelial differentiation of PMSCs with VEGF and bFGF for 10 days, while the exact proportion of differentiated ECs was unknown [34]. Given the superior angiogenic potential of PMSCs, there is great interest in evaluating the potential of endothelial differentiation in PMSCs with more stable and effective methods. In our study, under the same induction method previously reported by Wang et al. [7], approximately 70% differentiated ECs from PMSCs were obtained steadily on day 14. Hence, our results demonstrated that, compared to the BMMSCs counterparts, PMSCs might also have a superior potential for endothelial differentiation.

Undifferentiated MSCs prefer glycolysis instead of OXPHOS for energy metabolism [22] since anaerobic metabolism is beneficial to providing substrates for proliferation [54] and protect MSCs from aging [55, 56]. During MSCs differentiation, the energy acquisition pathway changes to meet the rapidly increased energy needs of the extensive biosynthesis, usually characterized by the upregulated aerobic metabolism and mitochondrial biogenesis [24, 25, 57]. In the BMMSCs osteogenic differentiation, OXPHOS is activated, while glycolytic activity is drastically reduced [25, 58]. However, Li et al. reported that adipogenic differentiation of BMMSCs exhibited increased activity in both OXPHOS and glycolysis [59], suggesting that the cellular metabolism changes during MSCs lineage commitment but with inconsistent results. The metabolic reprogramming in the endothelial differentiation of PMSCs has not been verified yet, which in this study was characterized by the augmented glycolysis and relatively suppressed OXPHOS with the mitochondrial disturbance. As far as we understand, there has been little study concerning the metabolism reprogramming in the endothelial differentiation of MSCs. Reportedly, activation of ERK1/2 signaling [60] and PI3K/AKT signaling [61] promote the endothelial differentiation of BMMSCs. Intriguingly, ERK1/2 signaling regulates the expression of glycolytic enzymes [62] and PI3K/AKT signaling suppresses the synthesis of glycogen and enhances glycolysis [63], which may be the circumstantial evidence, coupled with the Seahorse Cell Energy Metabolism analyses during BMMSCs endothelial differentiation in our study, to collectively indicated that glycometabolic preference for glycolysis is probably universal rather than specific during the endothelial differentiation. Besides, the glycometabolic shift direction of the endothelial differentiation of PMSCs is quite different from that in mesenchymal lineage differentiation, which may mainly owe to the metabolic nature of the ECs. ECs are glycolysis addicts as 85% of the ATP is produced glycolytically though exposed to the highest oxygen levels in the blood [64]. On the one hand, by relying on glycolysis, ECs produce ATP with faster kinetics [65] and save oxygen to supply perivascular cells [65]. On the other hand, mitochondria in ECs are mainly producers of metabolic intermediates rather than ATP [66]. Hence, the transformation of glycometabolism characteristics in our study further confirmed the decent differentiation of PMSCs into ECs. Reportedly, metabolic conversion may not only be a crucial feature in the differential process but also a vital modulatory factor. Research has confirmed that the activation of glycolysis and suppression of OXPHOS could inhibit both the osteogenic and adipogenic differentiation of MSCs [59, 67, 68]. The transition from glycolysis to OXPHOS is tightly coupled to neuronal differentiation from neural progenitor cells. However, activation of glycolysis leads to the failure of neuronal differentiation and cell death, indicating that metabolism reprogramming in the right direction may be essential for cell differentiation and cell fate determination [69]. However, the underlying mechanisms between glycometabolism reprogramming and PMSCs endothelial differentiation remain to be verified.

Genetics play crucial roles in the multi-directional differentiation of MSCs [70], leading to the different morphological structures, phenotypic characteristics, and biological functions [71]. The interaction of multiple DEGs and regulation in glycometabolism reprogramming may be involved in the PMSCs endothelial differentiation. By comparing the RNA-seq data within the endothelial differentiation of PMSCs, the DEGs identified displayed high enrichment in cell cycle regulation and metabolic pathways. During the development of cells, tissue, and organ, the processes of cellular differentiation and cell division are usually considered mutually exclusive, though coordination of cell cycle and differentiation must exist as cells have to undergo cell cycle arrest to allow differentiation [72]. Therefore, the elaborate modulation of the cell cycle by DEGs may be beneficial to the endothelial differentiation of PMSCs. Our RNA-Seq results further revealed many upregulated glycolysis genes and downregulated mitochondrial genes during the endothelial differentiation of PMSC. Thereinto, 6-phosphofructo-2-kinase/fructose-2,6-bisphosphatase (PFKFB) family, a group of powerful glycolytic regulators, accelerates glycolytic flux by promoting fructose-2,6-bisphosphate (F2,6P2) synthesis and facilitating the allosteric activation of 6-phosphofructo-1-kinase 1 (PFK-1) [73]. Of the PFKFB family, there are four isozymes encoded by four genes (PFKFB1–4). PFKFB3, also termed placental PFK-2 [41, 42], has the highest kinase activity than bisphosphatase activity (700-fold), thus most favoring the catalytic activity to glycolysis [74]. Reportedly, PFKFB3 is involved in the differentiation of macrophages, myofibroblasts, and cardiac progenitor cells [75–77]. In this study, PFKFB2, PFKFB3, and PFKFB4 are all significantly induced, indicating that the upregulated PFKFB3 represented the augmented glycolytic flux in the endothelial differentiation of PMSCs. Moreover, through the genetic inhibition of PFKFB3, we demonstrated that PFKFB3 is a crucial enzyme not only mediating the metabolism reprogramming but also modulating the endothelial differentiation of PMSCs. Hence, our results clarified that metabolic conversion and PMSCs endothelial differentiation may be inextricably intertwined.

It has been widely acknowledged that PFKFB3 participates in vasculogenesis and angiogenesis via regulating the glycolytic flux in ECs [64]. We have also reported that PFKFB3 plays a vital role in some pathophysiological changes in the placentas [36, 43], including the placental vasculature formation [44]. Besides, PFKFB3 modulates the glycolysis in pericytes to keep the stability and maturity of vessels [78]. Interestingly, PMSCs and pericytes may be inseparable and interconvert with each other in the vascular niches of placentas [45]. Therefore, we speculated that PFKFB3-induced glycometabolism reprogramming was very likely to not only modulate the endothelial differentiation but also further affect the angiogenic abilities of PMSCs. Reportedly, PMSCs can be recruited to sites of injury for repair [79]. Also, PMSCs can form capillary-like structures on Matrigel, while coculturing of PMSCs with ECs further augments capillary tube formation [80]. Intriguingly, compared to the undifferentiated state, induced PMSCs in our study showed enhanced migration, increased tube formation, and active engagement in the capillary tube formation with ECs both in vitro and in vivo. Additionally, PMSCs’ paracrine-mediated angiogenesis is certain, characterized by secreting a plethora of bioactive molecules to enhance tube formation and migration of ECs [81]. In this study, compared to the undifferentiated states, the culture medium of endothelium-differentiated PMSCs was more proangiogenic. Therefore, endothelial differentiation of PMSCs may be beneficial to therapeutic angiogenesis as well as vascular repair, while those effects were all suppressed via genetic inhibition of PFKFB3 in our study, indicating that PFKFB3-induced glycometabolism reprogramming could modulate the PMSCs endothelial differentiation and further affect the angiogenic abilities. In recent years, lab studies have shown promising results of using PMSCs in mice models of multiple ischemic diseases. Possibly by migration, direct differentiation, and incorporation [80, 81] or proangiogenic secretion [82], PMSCs augmented the formation of vessel network and enhanced the vessel density and perfusion at the ischemic sites [83, 84]. Although there remains the challenge of heterogeneity in the isolated cells, the growth rate and encouraging results of clinical trials (NCT03006770, NCT00919958, NCT00951210, NCT02264288 https://clinicaltrials.gov) using PMSCs for ischemic diseases further indicate the favor of PMSCs in clinical therapeutic angiogenesis [85]. In this case, our understanding of cellular glycometabolism and its regulatory effects on endothelial differentiation may improve the angiogenic potency of PMSCs in clinical therapeutic angiogenesis.

## Conclusion

Taken together, our study revealed that PMSCs possessed the superior potential of endothelial differentiation, accompanied by glycometabolism reprogramming. Moreover, through the transcriptome approach, glycolysis enzyme PFKFB3 was confirmed as a potential modulator for PMSCs endothelial differentiation and angiogenic capacities. Our study may propose and improve PMSCs as a putative strategy for clinical therapeutic angiogenesis.

## Supplementary Information

**Additional file 1: Fig S1**. Glycometabolism reprogramming during the endothelial differentiation of BMMSCs (**A**-**C**) The OCR assay was used to observe the basal and maximal mitochondrial respiratory function. (**D**-**F**) Glycolysis and glycolysis capacity was detected by ECAR assay. Data are shown as the mean ± SD from three independent experiments and the representative result is shown. *: P<0.05. **: P<0.01. ***: P<0.001 by Student’s t test. SD: standard deviation. EC-differentiated BMMSC: the induced BMMSCs group for endothelial differentiation.

## Data Availability

The data in this study are available from the corresponding author on reasonable request.

## Abbreviations

ECs: Endothelial cells
MSCs: Mesenchymal stem cells
PMSCs: Placenta-derived mesenchymal stem cells
BMMSCs: Bone marrow-derived mesenchymal stem cells
ATP: Adenosine triphosphate
OXPHOS: Oxidative phosphorylation
RNA-seq: Ribonucleic acid sequencing
PBS: Phosphate-buffered saline
HUVECs: Human umbilical vein endothelial cells
VEGF: Vascular endothelial growth factor
bFGF: Basic fibroblast growth factor
EGF: Epidermal growth factor
IGF: Insulin-like growth factor
qRT-PCR: Quantitative real-time quantitative polymerase chain reaction
vWF: Von Willebrand factor
VEGFR-2: Vascular endothelial growth factor receptor-2
OCR: Oxygen consumption rate
ECAR: Extracellular acidification rate
2-DG: 2-Deoxyglucose
MMP: Mitochondrial membrane potential
DEGs: Differentially expressed genes
BP: Biological process
CC: Cellular component
MF: Molecular function
PFKFB: 6-Phosphofructo-2-kinase/fructose-2,6-bisphosphatase
F2,6P2: Fructose-2,6-bisphosphate
