# Supplementary figures and images for: PFKFB3-mediated glycometabolism reprogramming modulates endothelial differentiation and angiogenic capacity of placenta-derived mesenchymal stem cells
Source: Stem Cell Res Ther. 2022 Aug 2;13:391. doi: 10.1186/s13287-022-03089-3 (PMC9344722; doi:10.1186/s13287-022-03089-3)

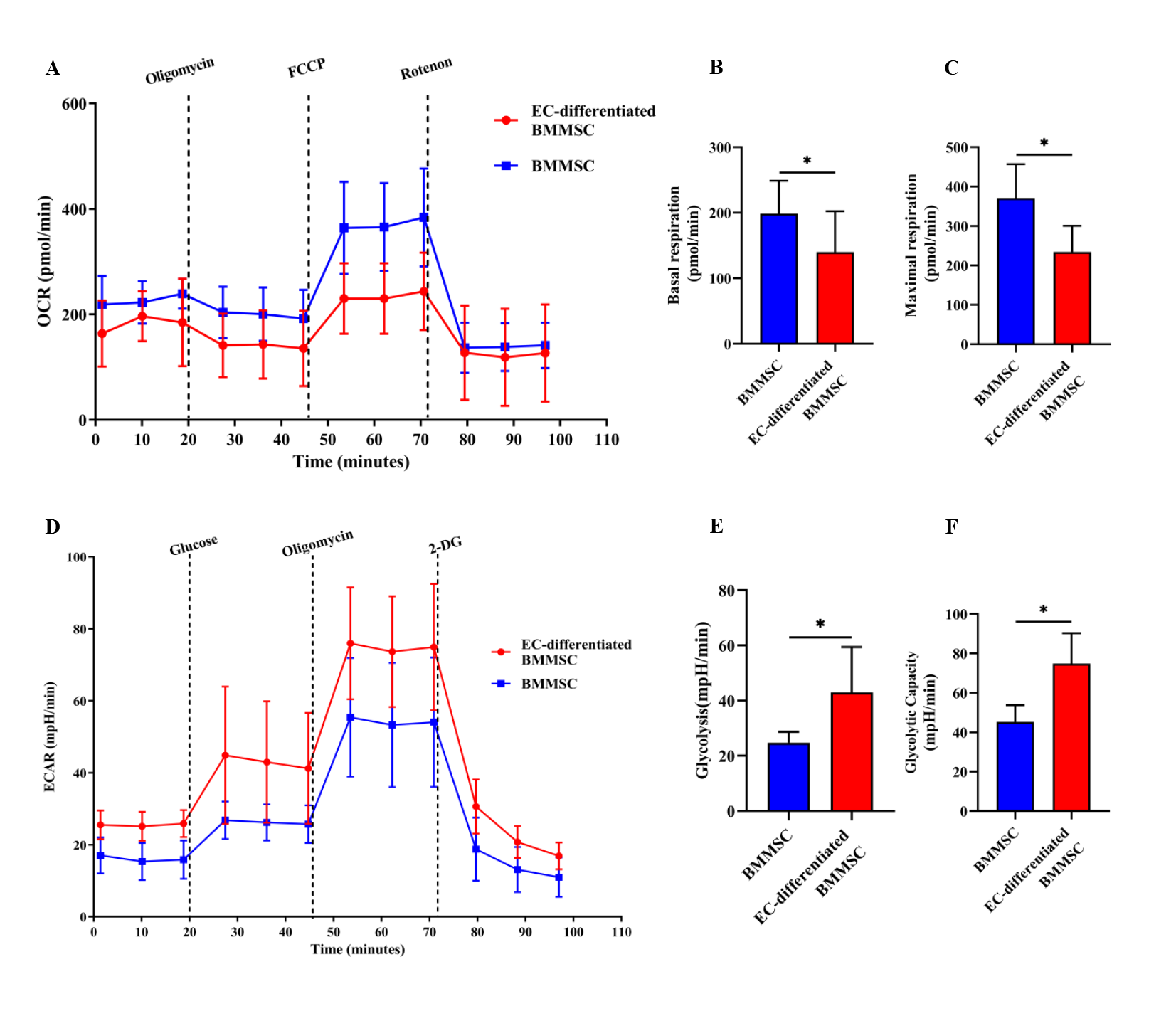

Supplement: Supplementary file 1 — Additional file 1: Fig S1. Glycometabolism reprogramming during the endothelial differentiation of BMMSCs (A-C) The OCR assay was used to observe the basal and maximal mitochondrial respiratory function. (D-F) Glycolysis and glycolysis capacity was detected by ECAR assay. Data are shown as the mean ± SD from three independent experiments and the representative result is shown. *: P<0.05. **: P<0.01. ***: P<0.001 by Student’s t test. SD: standard deviation. EC-differentiated BMMSC: the induced BMMSCs group for endothelial differentiation. [file 13287_2022_3089_MOESM1_ESM.tif]
